# Full automation of spinal stereotactic radiosurgery and stereotactic body radiation therapy treatment planning using Varian Eclipse scripting

**DOI:** 10.1002/acm2.13017

**Published:** 2020-09-23

**Authors:** Jose R. Teruel, Martha Malin, Elisa K. Liu, Allison McCarthy, Kenneth Hu, Bejamin T. Cooper, Erik P. Sulman, Joshua S. Silverman, David Barbee

**Affiliations:** ^1^ Department of Radiation Oncology Laura and Isaac Perlmutter Cancer Center NYU Langone Health New York NY USA

**Keywords:** spinal metastases, stereotactic body radiation therapy, stereotactic radiosurgery, treatment plan automation

## Abstract

The purpose of this feasibility study is to develop a fully automated procedure capable of generating treatment plans with multiple fractionation schemes to improve speed, robustness, and standardization of plan quality. A fully automated script was implemented for spinal stereotactic radiosurgery/stereotactic body radiation therapy (SRS/SBRT) plan generation using Eclipse v15.6 API. The script interface allows multiple dose/fractionation plan requests, planning target volume (PTV) expansions, as well as information regarding distance/overlap between spinal cord and targets to drive decision‐making. For each requested plan, the script creates the course, plans, field arrangements, and automatically optimizes and calculates dose. The script was retrospectively applied to ten computed tomography (CT) scans of previous cervical, thoracic, and lumbar spine SBRT patients. Three plans were generated for each patient — simultaneous integrated boost (SIB) 1800/1600 cGy to gross tumor volume (GTV)/PTV in one fraction; SIB 2700/2100 cGy to GTV/PTV in three fractions; and 3000 cGy to PTV in five fractions. Plan complexity and deliverability patient‐specific quality assurance (QA) was performed using ArcCHECK with an Exradin A16 chamber inserted. Dose objectives were met for all organs at risk (OARs) for each treatment plan. Median target coverage was GTV V100% = 87.3%, clinical target volume (CTV) V100% = 95.7% and PTV V100% = 88.0% for single fraction plans; GTV V100% = 95.6, CTV V100% = 99.6% and PTV V100% = 97.2% for three fraction plans; and GTV V100% = 99.6%, CTV V100% = 99.1% and PTV V100% = 97.2% for five fraction plans. All plans (n = 30) passed patient‐specific QA (>90%) at 2%/2 mm global gamma. A16 chamber dose measured at isocenter agreed with planned dose within 3% for all cases. Automatic planning for spine SRS/SBRT through scripting increases efficiency, standardizes plan quality and approach, and provides a tool for target coverage comparison of different fractionation schemes without the need for additional resources.

## 1 INTRODUCTION

Spinal metastases are devastating complications of malignant tumors that can decrease quality of life due to pain, impaired mobility, or neurologic disturbances.^1^, ^2^ Surgical resection has been the mainstay of treatment to reduce tumor burden and stabilize the surrounding anatomy, but may not be feasible in patients with poor performance status or tumors that abut critical organs.^3^ Radiation, now widely adopted for the treatment of spinal metastases, can be delivered as adjuvant to surgery, or as definitive treatment, to improve tumor and symptomatic control. The adoption of stereotactic immobilization and real‐time image guidance along with advances in treatment planning has allowed for treatment of spinal lesions using larger fraction sizes to improve tumor control and reduce treatment burden.^4^

RTOG 0631 phase II concluded that spine SRS was safe and feasible to be implemented in a high‐level cooperative group trial.^5^ Single institution studies demonstrate an advantage of using SRS/SBRT in terms of increased local control and complete response compared to conventional three‐dimensional conformal radiotherapy (3DCRT).^6^, ^7^, ^8^, ^9^, ^10^, ^11^, ^12^, ^13^ Sprave et al^14^ reported improved pain response on a randomized phase II trial evaluating single fraction spine SBRT vs 3DCRT. The SABR‐COMET randomized phase II trial compared standard 3DCRT palliative radiotherapy to SBRT for oligometastatic patients (including patients with spinal metastases) and reported a median overall survival of 28 months in the control group vs 41 months in the SABR group. Treatment‐related deaths occurred in 3 (none of which were spine) of 66 the patients after SABR, compared to none (n = 33) in the control group.^15^ The most feared complication from spine SRS is radiation myelopathy. Therefore, the dose to the spinal cord when performing spine SRS needs to be tightly controlled. A retrospective review on 1388 patients reported a 0.4% rate of myelophaty,^16^ and prospective studies have presented a range between 0 and 3%.^5^, ^17^, ^18^, ^19^ Other relevant toxicities associated with spine SRS treatments are vertebral compression fractures (VCF)^20^, ^21^, ^22^ and esophageal toxicity (fistula, ulcer, stenosis).^23^, ^24^

Current approaches to spine SRS/SBRT require generation of complex treatment plans using intensity‐modulated radiotherapy (IMRT) and volumetric modulated arc therapy (VMAT) treatment delivery techniques. Spine SRS/SBRT targets are often adjacent to the spinal cord and esophagus, both organs at risk (OARs) where overdose can lead to unacceptable complications such as myelopathy and esophageal perforation.^25^, ^26^ In order to achieve adequate dose coverage on the target while limiting the dose to OARs, spine SRS treatment plans must have steep dose gradients. Additionally, the high dose region must often have a concave shape to align to the spinal canal/vertebral body interface. The resulting treatment plans are often highly modulated and can push the limits of the accuracy of the treatment planning system. One study found that almost 15% of treatment plans were unfit for clinical use as they failed patient‐specific quality assurance (QA).^27^

Various studies have explored ways to improve the planning techniques used in spine SRS/SBRT planning.^27^, ^28^, ^29^ Multiple groups have compared IMRT and VMAT planning approaches with the typical finding that VMAT‐based plans improved PTV coverage over IMRT approaches.^28^, ^29^, ^30^ Several studies have evaluated specific technical factors in the planning process. Snyder et al.^28^ evaluated the use of jaw tracking and the impact of different energies on dosimetric metrics. Ayala et al.^27^ found that plan quality could be retained, treatment efficiency increased, and IMRT QA pass rates improved by limiting the number of segments per beam and increasing the minimum area per segment for step‐and‐shoot spine SRS plans. Different planning and treatment delivery systems used for spine SRS were compared by Moustakis et al.^30^ Finally, Saenz et al. evaluated a spine‐specific treatment planning system with a new optimization algorithm developed to overcome some of the limits found in traditional multipurpose planning systems.^31^

The optimal dose and fractionation scheme for spine SRS/SBRT is debated with widely varying approaches in clinical practice.^32^ If planning goals for one fractionation scheme cannot be adequately met, one approach is to change the fractionation to improve tumor coverage. This patient‐specific exploratory treatment planning to evaluate multiple fractionation schemes is time consuming and often clinically unfeasible in the setting of manually generated treatment plans. As the burden of spinal metastases increases secondary to the longer life expectancy of cancer patients, there is a growing need for efficient and accurate treatment planning in spine SRS.

Automated and semiautomated treatment planning approaches have the potential to produce high‐quality clinical plans while reducing the time spent on treatment optimization, allowing for unsupervised optimization and calculation, therefore decreasing the planning time. Exploratory demonstrations in head and neck,^33^ breast,^34^, ^35^ and other sites^36^, ^37^, ^38^ have produced results comparable to corresponding clinical plans. However, most studies focused on optimization approaches alone, which does not account for the complete treatment planning process. In addition, the translation of those results to the spine is challenging. An application of knowledge‐based planning has been explored for spine tumors, but cannot be used to evaluate multiple fractionation schemes.^39^ There remains a critical need to generate multiple high‐quality plans for different fractionation schemes in a clinically feasible manner.

The purpose of this feasibility study is to develop a fully automated procedure, using a widely available, all‐purpose treatment planning software capable of generating treatment plans with multiple fractionation schemes to improve speed, robustness, and standardization of plan quality. Using only a limited number of user inputs, automated treatment plans can be generated to aid in clinical decision‐making and to decrease the required treatment planning time.

## 2 MATERIALS AND METHODS

### 2.A Subjects

Ten previously treated spine SBRT patients from our institution were anonymized and retrospectively replanned using the automatic treatment planning script. The patient dataset included targets treating the cervical (2), thoracic (7), and lumbar (1) spine regions. CT simulation was performed on a Siemens SOMATOM scanner (Siemens, Erlangen, Germany) with the following scan protocol: 1.5 mm slice thickness, 500 mm acquisition diameter, and extended field of view (FOV) reconstruction of 650 mm. The use of anonymized retrospective CT scans for dosimetry studies was approved by our Internal Review Board (IRB S18‐00659).

### 2.B Treatment planning script

**Fig. 1:**
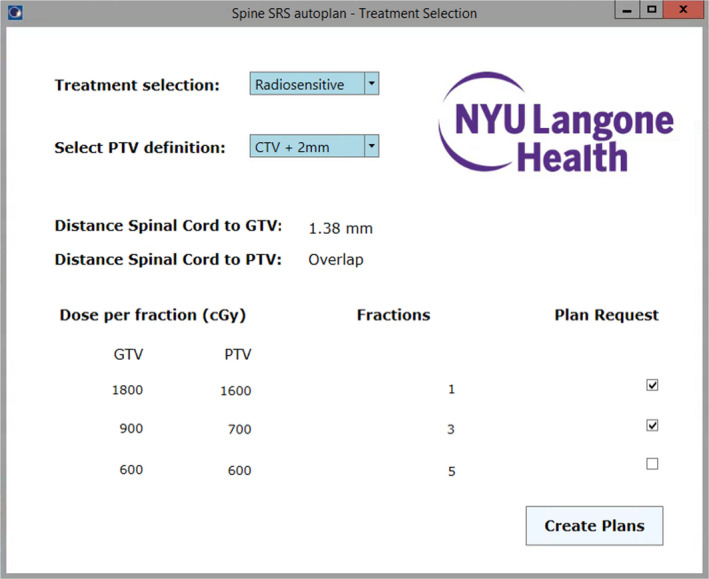
Graphic user interface for automatic planning script. The user is presented with options for treatment selection and planning target volume generation, information regarding distance between spinal cord and targets, as well as the ability to revise fractionation and select requested plans.

A fully automated script for spine SRS/SBRT plan generation, optimization, and calculation was developed using the Eclipse scripting application programing interface (ESAPI) v15.6 (Varian Medical Systems, Palo Alto, CA). The script was written in C# using the Visual Studio environment (Microsoft, Redmond, WA), Windows presentation foundation (WPF), and the Eclipse API binary libraries provided with Eclipse v15.6. The autoplanning script requires completed structure delineation, including all organs at risk (OARs), plus the gross tumor volume (GTV), and clinical target volume (CTV). Gross tumor volume and CTV were contoured following consensus guidelines for spine SRS.^23^, ^40^ For all these cases, the spinal cord was contoured using either magnetic resonance imaging (MRI), CT myelogram, or both. Initial script execution presents a graphic user interface (GUI) displaying treatment options and information (Fig. 1). The script GUI allows the user to select a prescription template (radiosensitive or radioresistant), and three options for PTV generation: (a) PTV = CTV; (b) PTV = CTV plus a 1‐mm isotropic margin; (c) PTV = CTV plus a 2‐mm isotropic margin. Information regarding distance or overlap between spinal cord and targets is provided to assist with clinical decision‐making. This metric is presented using two approaches: Shortest distance between spinal cord and GTV/PTV, and shortest distance between spinal cord and GTV/PTV centroid. Finally, for the prescription, template selected the script offers the user the option to select three different fractionations and proceed with the execution.

For each requested plan, the script creates the course, plans, beam arrangement, optimization structures, digitally reconstructed radiographs (DDRs), and automatically optimizes and calculates dose. Optimization structures included high‐resolution structures forcing the dose fall‐off between targets and the most proximal organs at risk, such as the spinal cord and the esophagus, to ensure that there are no conflicting objectives. Additional optimization structures included rings for dose fall‐off control and structures to define dose decrease between SIB dose level and the lower dose target region that should receive complete coverage. Priority is given to meet the maximum dose constraint to spinal cord and esophagus. The script loads the required optimization objectives based on the structures present in the structure set, including the following structures (if present): GTV, CTV, PTV, spinal cord, cauda equina, esophagus, brachial plexus, heart, trachea, skin, small bowel, stomach, colon, rectum, kidneys, lungs, and sacral plexus. At a minimum GTV, CTV and spinal cord (and/or cauda equina if lumbar spine) have to be present for the script to run. If any of these structures are not contoured, an error is launched that alerts the user. The PTV is automatically contoured by the criteria selected by the user in the GUI as specified in the preceding section. Additionally, planning organ at risk volumes (PRVs) for spinal cord and cauda equina are created using a 2‐mm expansion.

Finally, the script generates dose verification plans for SNC ArcCHECK (Sun Nuclear, Melbourne, FL), exports the plan to Mobius (Varian) and RadCalc (Lifeline, Austin, TX) for monitor units (MUs) check, and creates a dose objectives summary document for each plan.

### 2.C Treatment plans

For this study, three plans were automatically generated for each patient according to our radiosensitive de novo prescription scheme that included the following: (a) simultaneous integrated boost (SIB) plan with 1800 cGy/1600 cGy dose to GTV/PTV in one fraction; (b) SIB 2700 cGy/2100 cGy dose to GTV/PTV in three fractions; (c) 3000 cGy dose to PTV in five fractions. The use of SIB for single or three fractions treatments has been reported previously and used for this retrospective feasibility study.^41^, ^42^ Dose objectives for each fractionation are reported in Tables 1 and 2 and are based on previous evidence to minimize rate of toxicities.^5^, ^25^, ^43^, ^44^, ^45^ All treatment plans were calculated for delivery on a Varian Edge treatment machine equipped with high‐resolution multileaf collimator (HD120 MLC). All plans used the same field arrangement of four co‐planar full arcs with the isocenter automatically located at the GTV centroid and four different collimation rotations: 4°, 356°, 7°, 353°. Additional settings include the use of 10X flattening filter‐free (FFF) energy, analytic anisotropic algorithm (AAA), photon optimizer (PO) and a 0.125‐cm calculation resolution. Optimization convergence mode was set to “on” for all cases. Intermediate dose calculation and normal tissue objective were employed for optimization.

**Table 1:** Dose objectives and results for single fraction and three fraction plans generated automatically for ten cases.

Structure	Dose objective (1fx)	Result (1fx)	Dose objective (3fx)	Result (3fx)
GTV (n = 10)	V100% ≥ 95%	87.3% (76.2%, 97.2%)	V100% ≥ 95%	95.6% (89.1%, 99.3%)
	Dmax < 130%	122.3% (114.1%, 130.3%)	Dmax < 130%	124.8% (111.0%, 130.2%)
CTV (n = 10)	V100% ≥ 90‐80%	95.7% (79.7%, 100%)	V100% ≥ 90‐80%	99.6% (97.9%, 100.0%)
PTV (n = 10)	V100% ≥ 90‐80%	88.0% (74.9%, 100%)	V100% ≥ 90‐80%	97.2% (92.9%, 95.6%)
Spinal Cord (n = 9)	D0.035cc < 1000cGy	934.0 (808.3, 979.1) cGy	D0.035cc < 2190cGy	1313.8 (1009.2, 1765.0) cGy
	V800cGy < 1cc	0.470 (0.054, 0.919) cc	V1230cGy < 1.2cc	0.105 (0.0, 0.537) cc
Spinal Cord + 2mm (n = 9)	D0.035cc < 1200cGy	1079.9 (938.0, 1177.4) cGy	D0.035cc < 2390cGy	1846.2 (1603.3, 2080.5) cGy
	V1000cGy < 1cc	0.328 (0.0, 0.992) cc	V1430cGy < 1.2cc	0.451 (0.197, 0.936) cc
Esophagus (n = 8)	D0.035cc < 1700cGy	1294.4 (549.3, 1619.3) cGy	D0.035cc < 2520cGy	1737.8 (646.2, 2277.0) cGy
	V1200cGy < 1cc	0.249 (0.0, 0.712) cc	V1770cGy < 5cc	0.006 (0.0, 0.742) cc
Heart (n = 5)	D0.035cc < 2200cGy	689.1 (231.5, 1318.8) cGy	D0.035cc < 3000cGy	923.6 (276.8, 1831.6) cGy
	V1600cGy < 15cc	0.0 (0.0, 0.0) cc	V2400cGy < 15cc	0.0 (0.0, 0.0) cc
Skin (n = 8)	D0.035cc < 1600cGy	758.8 (487.9, 1066.0) cGy	D0.035cc < 3300cGy	985.3 (684.4, 1391.8) cGy
	V1400cGy < 10cc	0.0 (0.0, 0.0) cc	V3000cGy < 10cc	0.0 (0.0, 0.0) cc
Lungs (n = 6)	MVS700cGy > 1000cc	2207.0 (1886.4, 3228.2) cc	MVS1160cGy > 1500cc	2262.7 (1895.0, 3238.0) cc
Liver (n = 4)	MVS910cGy > 700cc	1882.9 (887.6, 3428.2) cc	MVS1920cGy > 700cc	1882.9 (911.1, 3428.2) cc
Trachea (n = 3)	D0.035cc < 2020cGy	1086.5 (860.1, 1467.1) cGy	D0.035cc < 3000cGy	1436.8 (1166.8, 2057.9) cGy
	V880cGy < 4cc	0.892 (0.012, 3.202) cc	V1500cGy < 4cc	0.005 (0.0, 1.932) cc
Brachial Plexus, Right (n = 2)	D0.035cc < 1600cGy	1547.1 (1546.2, 1548.0) cGy	D0.035cc < 2400cGy	2211.6 (2198.5, 2224.7) cGy
	V1400cGy < 3cc	0.440 (0.294, 0.585) cc	V2040cGy < 3cc	0.173 (0.154, 0.192) cc
Brachial Plexus, Left (n = 2)	D0.035cc < 1600cGy	1498.6 (1449.2, 1548.0) cGy	D0.035cc < 2400cGy	2125.3 (2057.1, 2193.4) cGy
	V1400cGy < 3cc	0.205 (0.059, 0.350) cc	V2040cGy < 3cc	0.138 (0.041, 0.235) cc
Cauda Equina (n = 1)	D0.035cc < 1600cGy	1177.8 cGy	D0.035cc < 2400cGy	2050.9 cGy
	V1200cGy < 1cc	0.009 cc	V2190cGy < 5cc	0.0 cc
Cauda Equina + 2mm (n = 1)	D0.035cc < 1800cGy	1424.6 cGy	D0.035cc < 2600cGy	2409.1 cGy
	V1400cGy < 1cc	0.057 cc	V2390cGy < 5cc	0.054 cc

Median values with range (minimum, maximum) in parentheses.

**Table 2:** Dose objectives and results for five fraction plans generated automatically for ten cases.

Structure	Dose objective	Result
GTV (n = 10)	V100% ≥ 95%	99.6% (96.1%, 100%)
CTV (n = 10)	V100% ≥ 90‐80%	99.1% (96.7%, 100%)
PTV (n = 10)	V100% ≥ 90‐80%	97.2% (88.9%, 99.4%)
	Dmax < 130%	128.6% (110.5%, 130.0%)
Spinal Cord (n = 9)	D0.035cc < 3000cGy	2423.6 (1711.0, 2691.3) cGy
	D0.35cc < 2300cGy	1875.4 (1364.8, 2027.7) cGy
	D1.2cc < 1450cGy	1087.5 (269.3, 1194.8) cGy
Spinal Cord + 2mm (n = 9)	D0.035cc < 3000cGy	2847.7 (2554.5, 2878.1) cGy
Cauda Equina (n = 1)	D0.035cc< 3200cGy	2886.6 cGy
	D5cc < 3000cGy	159.7 cGy

Median values with range (minimum, maximum) in parentheses.

### 2.D Patient‐specific QA

To evaluate deliverability of the treatment plans, patient‐specific QA was performed for all 30 individual plans (ten treatment plans for each fractionation scheme). Dose verification plans were created for a SNC ArcCHECK diode array device and evaluation was carried out using the composite plan. Global gamma metric passing rates were evaluated using three different threshold levels — 3% at 2 mm, 2% at 2 mm, and 3% at 1 mm, all with a 10% threshold dose.

### 2.E Ion chamber measurement

For each patient‐specific QA measurement, the dose to the center of the ArcCHECK was measured with an Exradin A16 micro ion chamber (Standard Imaging, Middleton, WI) that was inserted in the ArcCheck using the appropriate acrylic holder. The deviation between the dose in the Eclipse treatment plan (TP) and the dose delivered to the ion chamber (IC) was calculated as:

$$\text{Dose}\;\text{deviation}\left(\%\right)=100\frac{\left|{\text{Dose}}_{\text{IC}}‐{\text{Dose}}_{\text{TP}}\right|}{{\text{Dose}}_{\text{TP}}}$$

To convert the charge reading to dose, the average of five 100 MU open field measurements (10 × 10 cm) with the ion chamber at isocenter inserted in the ArcCHECK was referenced to the dose reported in Eclipse for the same open beam setting.

## 3 RESULTS

The location of the spinal tumors used in this study encompassed the cervical, thoracic, and lumbar spine. Lesions were located in C6, C6/C7 (continuous GTV contour), T2/T4 (individual GTV contours and combined CTV), T6, T7, T9, T10, T11, and L3. The median GTV, CTV, and PTV volumes were 5.3, 30.3, and 50.3 cm^3^, ranging between 0.8 and 43.7 cm^3^, between 9.6 and 54.6 cm^3^, and between 18.2 and 82.8 cm^3^ respectively. The majority of the automated tasks (plan creation, beam setup, optimization structure creation, and optimization template loading) were completed in <5 s. Using the settings indicated in the methods, a complete run from start to finish including the generation of three treatment plans with different fractionation was completed in under 2 h, with each plan taking approximately 30–40 min.

**Fig. 2:**
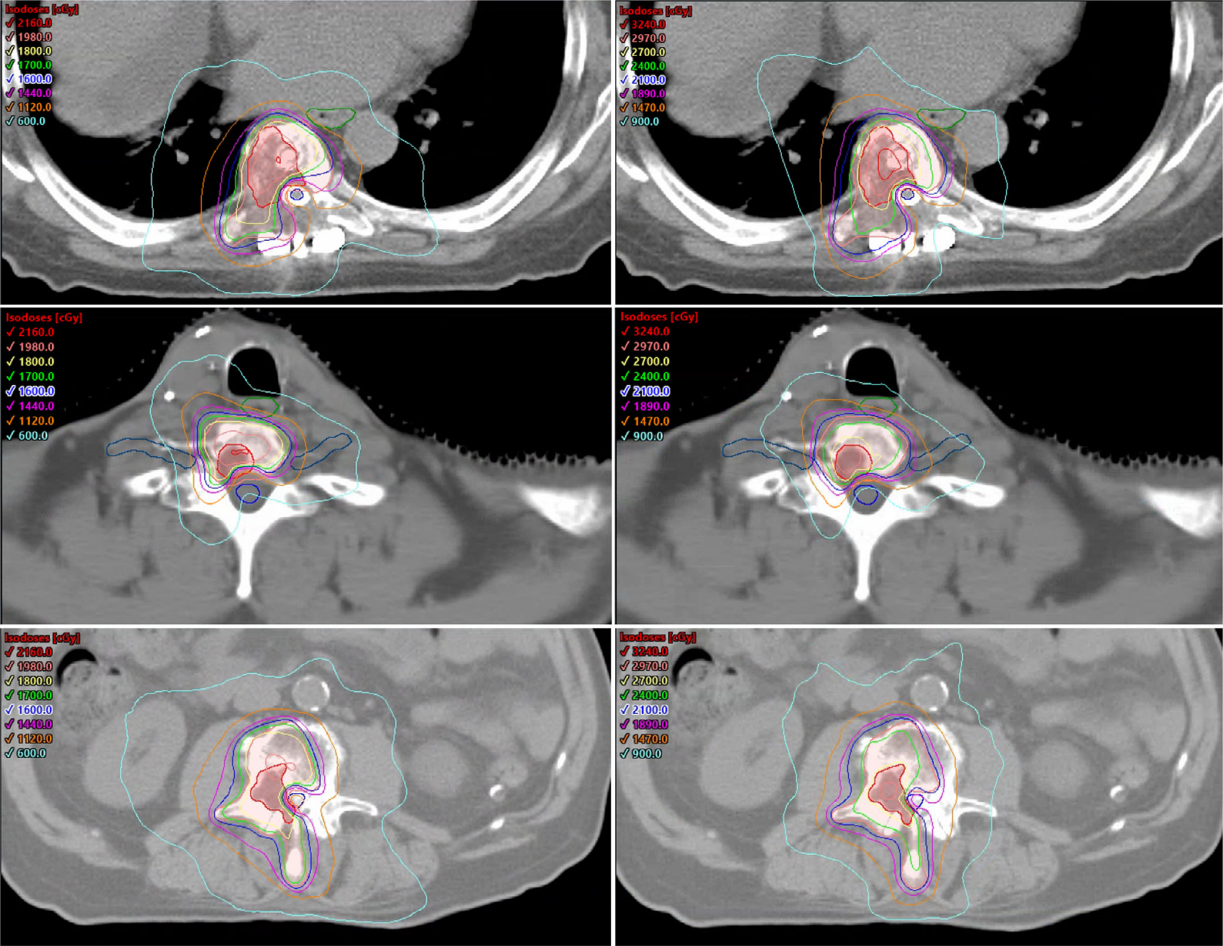
Axial view for three SRS treatment plans. Left: Single fraction plans [1800 cGy to gross tumor volume (GTV), 1600 cGy to planning target volume (PTV)]. Right: Three fraction plans (2700 cGy to GTV, 2100 cGy to PTV). Top: Thoracic spine case. Middle: Cervical spine case. Bottom: Lumbar spine case. GTV as red segment. PTV as pink segment. Spinal cord, brachial plexus, and cauda equine as blue contours. Esophagus as green contour.

Single fraction and three fraction plans for one cervical, one thoracic, and one lumber case are presented side by side in Fig. 2. Dose objectives and results for single fraction and three fraction plans including targets and OARs are summarized in Table 1. A summary of dosimetric results for five fraction plans including targets, spinal cord, and cauda equina are presented in Table 2. Dose objectives were met for all OARs for each treatment plan including the three possible fractionations. The highest level of target coverage was obtained for five fractions plans. Three fraction plans met all target coverage constraints in all scenarios with the exception of the GTV due to the high dose required in the SIB prescription schema. As expected, single fraction plans produced the biggest compromise in target coverage due the largest difference between the dose that the OARs were allowed to receive, particularly the spinal cord, and the prescription dose to the targets.

**Fig. 3:**
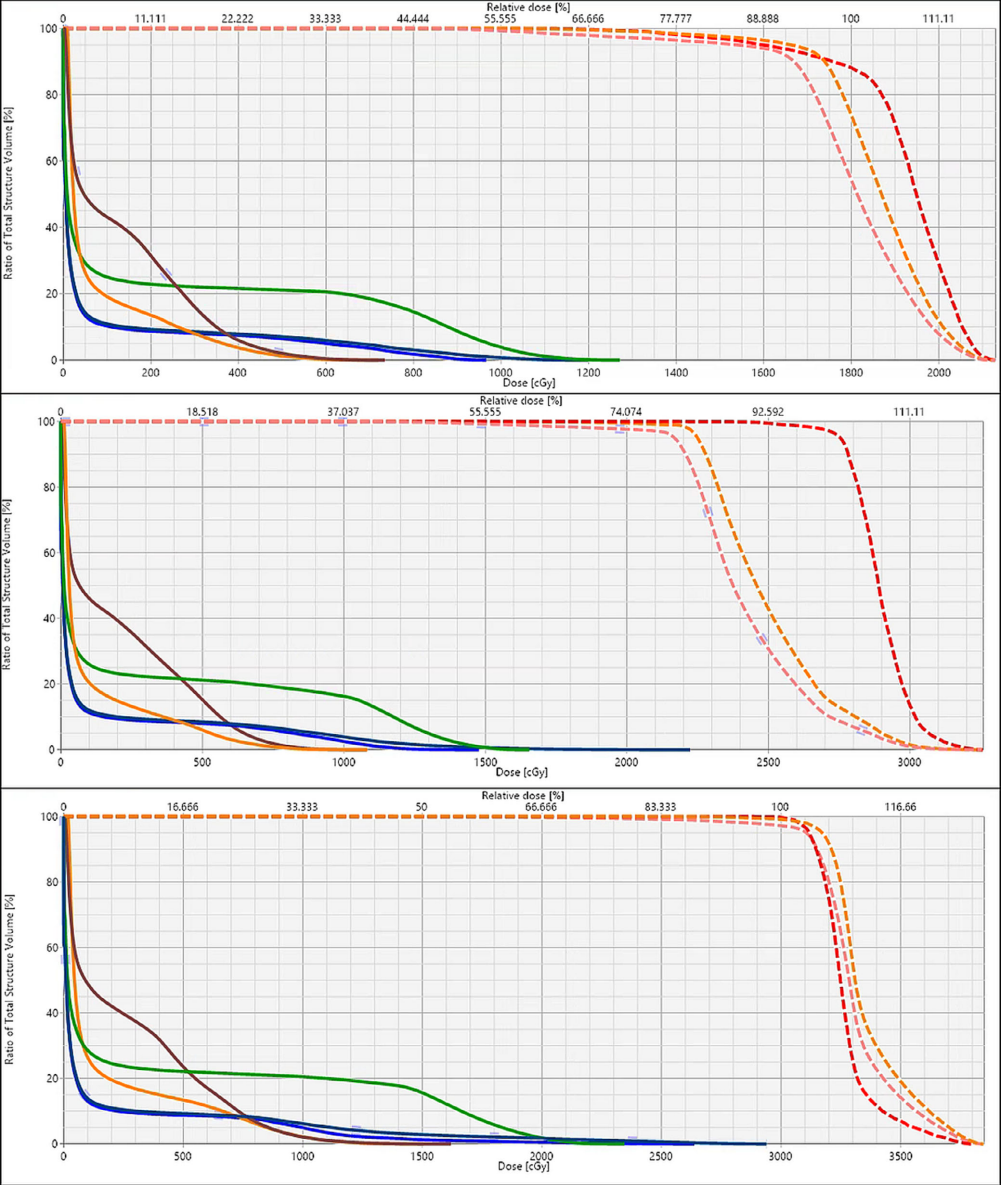
Dose volume histogram (DVH) for a thoracic spine case (T10). DVH includes gross tumor volume (GTV) (red dashed), clinical target volume (orange dashed), planning target volume (PTV) (pink dashed), spinal cord (blue), spinal cord + 2mm (dark blue), esophagus (green), heart (orange), and liver (brown). Top: Single‐fraction plan (SIB 1800/1600 cGy to GTV/PTV). Middle: Three fractions plan (SIB 2700/2100 cGy to GTV/PTV). Bottom: Five fractions plan (3000 cGy to the PTV).

The dose volume histograms for one case and the three different fractionations are presented in Fig. 3. The median PTV Conformity Index using the 100% isodose line (CI 100%) was 1.16 for single fraction plans, 1.20 for three fraction plans, and 1.18 for five fraction plans. For one of the cases only, PTV CI 100% was 1.4 or higher (1.40 for the three fractions plan and 1.42 for the five fractions plan). Among the three fractionations, single fraction plans had the lowest amount of MUs per cGy. Median number of MUs/cGy was 2.91, 3.29, and 3.56 for single fraction, three fractions, and five fractions plan respectively. The ranges and total number of MUs is reported in Table 3. The beam‐on time for delivery was below 6 min for all cases.

**Table 3:** Total number of monitor units (MUs) and MUs/cGy for different fractionations.

	Total Monitor Units (MUs)	MUs/cGy
Single fraction	5234 (4321, 6784) MUs	2.91 (2.40, 3.77)
Three fractions	2960 (2623, 2474) MUs	3.29 (2.91, 3.86)
Five fractions	2138 (1961, 2555) MUs	3.56 (3.27, 4.26)

Median value with range (minimum, maximum) in parentheses.

For simultaneous integrated boost (SIB) plans (single and three fractions), the cGy value is based on the highest dose level.

For single fraction plans, the patient‐specific QA median pass rate was 99.0% (lowest of 96.2%), 97.0% (lowest of 91.8%), and 94.5% (lowest of 91.2%) when using a criterion of 3%/2 mm, 2%/2 mm, and 3%/1 mm respectively. For three fraction plans, the median pass rate was 97.5% (lowest of 94.2%), 95.4% (lowest of 90.7%), and 92.9% (lowest of 89.0%), respectively. For five fraction plans, the median pass rate was 97.8% (lowest of 95.8%), 94.9% (lowest of 90.7%), and 93.7% (lowest of 88.1%), respectively.

## 4 DISCUSSION

Our results demonstrate that it is feasible to establish an automatic workflow to create high‐quality treatment plans for spine SRS. The tool presented in this study automated plan creation, optimization, and calculation with the primary goal of meeting all OARs constraints while maximizing target coverage irrespective of lesion location, size, distance, and fractionation. Target coverage varied depending on the need to spare the most critical OARs, such as spinal cord, cauda equina, esophagus, trachea, and brachial plexus that, for some cases, are located adjacent to spinal metastases or even overlap with the PTV. As expected, the target coverage increased when the fractionation was increased due to the increased percent of the prescription dose allowed to be delivered to each OAR. The ability to create different fractionation plans provides an excellent tool to balance target coverage with the most optimal fractionation scheme.

All the plans presented here were obtained with only one optimization iteration, including one intermediate dose calculation, and without additional manual input in optimization. This approach was chosen in order to explore the feasibility of full plan automation; however, there is no reason why this tool could not be used in conjunction with a final manual input or a multicriteria optimization tool (MCO). One scenario we could envision in the use of this tool is an initial automatic multiplan generation, and subsequently, a final manual iteration for the plan with the fractionation selected. The main goals accomplished in our study rely on eliminating the time and effort required for plan creation, beam setup, optimization structure creation, creating/loading the appropriate optimization objectives pertinent to the lesion location on the spine, and setup optimization and calculation for multiple plans. In addition, due to the automatic nature of the process the time for creation of a large number of optimization structures that can be especially time consuming manually, becomes negligible. This is one of the script’s main features in order to force the steep gradient from the spinal cord limiting dose as effectively as possible while approaching the targets. The code is set up to evaluate the distance between dose limiting OARs and the target. Based on that distance and the dose objectives, a different number of optimization structures are created for each plan with the goal of maintaining the OARs constraint while also establishing an approximate 2 Gy per mm gradient to optimize target coverage. Therefore, the creation of optimization structures can be considered a customized process (not the same for every case) that is based on the distance between the OARs and the targets, and the dose difference that needs to be achieved. To achieve the same process manually becomes very time consuming and subject to observer variability. Total execution time is highly driven by the optimization/calculation settings including structure resolution, dose grid, convergence mode setting, etc. Therefore, the numbers reported should be carefully considered as they might vary depending on individual institutional practices. A key aspect of timing is that some of the typically very time consuming manual tasks, such as creation of optimization structures, setting up plans, fields and DRRs, loading or creating optimization objectives are reduced to just a few seconds. Additionally, the script can be run in stand‐alone mode without supervision (overnight for instance) and automatically saves the results.

Spine SRS treatment planning is labor intensive, requiring complex arrangements of multiple intensity modulated beams to deliver high doses per fraction in an effective and safe manner. This can impede the use of spine SRS for patients requiring urgent or emergent management who otherwise would be candidates. In addition, there is not a clear consensus as to which fractionation scheme should be employed for spine SRS treatments,^32^ and, in general, it would be very time consuming to create several plans manually for different fractionation schemes. Automatic treatment planning is a topic of increased interest for different sites to reduce treatment planning times and increase robustness and standardization.^33^, ^36^, ^37^, ^38^ However, most studies have focused on plan optimization, using either knowledge‐based techniques, automatic iterative optimization, or multicriteria optimization approaches. In this study, we have included scripting into our planning approach to automate the process of treatment planning after normal structure and target contouring. Furthermore, scripting is compatible with any optimization approach and knowledge‐based planning and MCO can be easily incorporated into the script. Finally, our tool allows the user to select the number of plans to be generated and the planning criterion to be used. This provides a useful tool for decision‐making.

Some limitations to, and decisions made in, this work need to be acknowledged. First, the current state of the software used for automatic planning only provides options for de novo cases. However, it is not uncommon for patients undergoing spine SRS/SBRT to have already received palliative treatment at an earlier point in time.^46^ Currently, we are working to incorporate that functionality, allowing the user to override the default dose limit to the spinal cord in terms of maximum dose allowed for the current plan or in terms of a combined biologically equivalent dose (BED). Second, the field setup using four co‐planar full arcs and the mentioned collimation rotations, while used in this study and found to provide excellent dose shaping, is not a requirement. Eclipse scripting allows the user to set every single parameter of the field arrangement (isocenter location, couch rotation, collimator rotation, arc rotation), and therefore, the field arrangement can be set automatically as desired using the scripting code. Third, it is important to acknowledge that the treatment plan time presented is an approximation. Fourth, for this study we added together cervical, thoracic, and lumbar spine cases due to the small sample size. The script is able to handle any location and consider any set of OARs contoured for planning. However, different locations present different challenges in terms of planning and influence the overall results reported in this study compared to a stratified analysis based on lesion location. Finally, due the retrospective nature of this project and the limited sample size, the performance of the script and quality of newly generated plans will need to be closely monitored and further validated when used to generate treatment plans for clinical use.

Future developments can be incorporated into our current automatic tool, thanks to the high degree of customization available using scripting. We are currently working on making the tool compatible with re‐irradiation treatments. We are also working to incorporate into the script a plan complexity calculation that can be used to evaluate how modulated/complex the plan is even prior to performing patient‐specific QA. Furthermore, a knowledge‐based planning model is currently in development for incorporation into the automatic optimization step, as previously suggested. Finally, the script can be further customized to employ and compare different beam arrangement configurations than the ones presented in this study.

## 5 CONCLUSION

Automatic treatment planning for spine SRS/SBRT using scripting can facilitate the task of planning for spine metastases. Automatic planning through scripting increases efficiency, standardizes plan quality and approach, and provides a tool for target coverage comparison of different fractionation schemes without the need for additional resources.

## CONFLICTS OF INTEREST

Honorarium from Varian Medical Systems (Jose Teruel) not directly related to the content of this manuscript.
